# Finding smoking hot‐spots: a cross‐sectional survey of smoking patterns by housing tenure in England

**DOI:** 10.1111/add.14544

**Published:** 2019-01-20

**Authors:** Sarah E. Jackson, Cheryll Smith, Hazel Cheeseman, Robert West, Jamie Brown

**Affiliations:** ^1^ Department of Behavioural Science and Health University College London London UK; ^2^ Action on Smoking and Health London UK

**Keywords:** Cessation support, housing, prevalence, quit attempts, smoking, smoking cessation

## Abstract

**Aims:**

To examine smoking prevalence, motivation and attempts to stop smoking, markers of cigarette addiction and success in quit attempts of people living in social housing in England compared with other housing tenures.

**Design and setting:**

A large cross‐sectional survey of a representative sample of the English population conducted between January 2015 and October 2017.

**Participants:**

A total of 57 522 adults (aged ≥ 16 years).

**Measurements:**

Main outcomes were smoking status, number of cigarettes per day, time to first cigarette, exposure to smoking by others, motivation to stop smoking, past‐year quit attempts and use of cessation support. Covariates were age, sex, social grade, region and survey year.

**Findings:**

Adults in social housing had twice the odds of being smokers than those living in other housing types [odds ratio (OR) = 2.09, 95% confidence interval (CI) = 1.98–2.22, *P* < 0.001]. Smokers in social housing consumed more cigarettes daily (adjusted mean difference = 1.09 cigarettes, 95% CI = 0.72–1.46, *P* < 0.001) and were more likely to smoke within 30 minutes of waking (OR = 1.63, 95% CI = 1.48–1.79, *P* < 0.001) than smokers living in other housing types. Prevalence of high motivation to stop smoking was similar across housing types (OR = 1.04, 95% CI = 0.91–1.19, *P* = 0.553). The prevalence of quit attempts and use of cessation support within the past year were greater in social compared with other housing (OR = 1.14, 95% CI = 1.03–1.26, *P* = 0.011; OR = 1.30, 95% CI = 1.09–1.54, *P* = 0.003), but success in quitting was much lower (OR = 0.57, 95% CI = 0.45–0.72, *P* < 0.001).

**Conclusions:**

In England, living in social housing is a major independent risk factor for smoking. These easily identifiable hot‐spots consist of smokers who are at least as motivated to stop as other smokers, but find it more difficult.

## Introduction

Smoking increases the risk of a range of diseases and is a leading preventable cause of early death and disability in every world region, with approximately one in seven adults smoking every day 1, 2, 3, 4. Prevalence in many countries, including the United Kingdom, has fallen markedly since the 1970s 5, 6, but to a lesser extent in those with greater social disadvantage 7, 8, 9. The need to address this increasing disparity has been recognized in many countries 10, 11. The 2017 tobacco control plan for England called for targeted action to address this inequality 12. High smoking prevalence among people living in social housing could be an important focus. In 2016, the UK Office of National Statistics reported that 33% of those who lived in social housing were cigarette smokers compared with approximately 10% of those who owned their home 13. However, it is not clear to what extent this high smoking rate stemmed from other factors; nor is it clear how far it reflects lower motivation to quit among smokers, greater difficulty quitting or both. This study examined adjusted smoking prevalence, and smoking and quitting characteristics of people living in social housing compared with other housing tenures.

There is substantial evidence linking housing to health inequalities through three main pathways: internal housing conditions, area characteristics and housing tenure 14. From a policy perspective, social housing could be a particularly important indicator in its own right 15. Social housing in England is let at lower rents on a secure basis to those who are most in need or struggling with their housing costs. Accommodation is owned and managed by registered providers, typically local authorities (local councils made up of publicly elected councillors) or housing associations (independent, not‐for‐profit organizations), and regulated and funded by the government. Because it is a location‐based indicator of ‘smoking hot‐spots’ (areas with high smoking prevalence) social housing could provide a highly tractable basis for targeted interventions. These could include localized anti‐smoking campaigns, provision of neighbourhood smoking cessation services and/or introduction of local smoke‐free policies. Interventions could capitalize on the tendency of smoking and smoking cessation to cluster in social networks 16. Broad behavioural science principles suggest that multi‐component interventions addressing local norms, providing action triggers and ensuring ready access to evidence‐based support for cessation could work synergistically with social interactions to create cultures of quitting, whereby social housing residents quit in unison, in identifiable localities 17, 18.

Probably the most controversial component of such an intervention would be to make social housing smoke‐free. There is a precedent for this, in that the US legislated to require all public housing developments to be smoke‐free by autumn 2018 19, although there are differences between the UK and US systems. If it were feasible to implement such a rule in the United Kingdom and it reduced the level of smoking in the home it could provide both a supportive environment for quitting smoking and mitigate the excess risk from passive smoking among non‐smokers, including children 20, 21, 22. This kind of policy would require clear evidence of benefit and have wide acceptability among residents. A better understanding of smoking rates directly linked to social housing, motivation to quit and difficulty quitting in this environment could contribute to this.

This paper addressed the following questions:

1. How does the prevalence of cigarette smoking in adults living in social housing compare with other housing tenures, adjusting for a range of socio‐demographic factors?
2. Among current smokers, how do the prevalence of high motivation to quit smoking and markers of cigarette addiction in adults living in social housing compare with other housing tenures, adjusting for a range of socio‐demographic factors?
3. Among past‐year smokers, how do the prevalence of a quit attempt in the past year and regular exposure to smoking by others in adults living in social housing compare with other housing tenures, adjusting for a range of socio‐demographic factors?
4. Among past‐year smokers who have made at least one quit attempt, how do the success rates and use of smoking cessation aids compare with other housing tenures, adjusting for a range of socio‐demographic factors?

## Methods

### Study design

This was a cross‐sectional national survey of a representative sample of adults (aged 16 years and older) in England. Smoking, smoking cessation and housing tenure data were collected in the Smoking Toolkit Study (STS) between January 2015 and October 2017 23.

#### Sampling

The survey uses a type of random location sampling, which is a hybrid between random probability and simple quota sampling 23, and included 57 522 respondents aged 16 years or older in England. Full details of the study's methods are available elsewhere, and comparisons with national data indicate that key variables such as socio‐demographics and smoking prevalence are nationally representative 23.

### Patient and public involvement

The wider toolkit study has been discussed with a diverse patient and public involvement (PPI) group, and the authors regularly attend and present at meetings at which patients and public are included. Interaction and discussion at these events help to shape the broad research priorities and questions. There is also a mechanism for generalized input from the wider public: each month interviewers seek feedback on the questions from all 1700 respondents, who are representative of the English population. This feedback is limited, and usually simply relates to understanding of questions and item options. No patients or members of the public were involved in setting the research questions or the outcome measures, nor were they involved in the design and implementation of this specific study. There are no plans to involve patients in dissemination.

### Ethics approval

Ethics approval for the STS was granted by the UCL Ethics Committee (ID 2808/005). All respondents provided informed verbal consent.

### Measures

#### Explanatory

Respondents whose homes belonged to a housing association or were rented from local authority were defined as ‘social housing’ residents. All other responses were categorized as ‘other housing’, which included houses which were ‘bought on a mortgage’, ‘owned outright by household’, ‘rented from private landlord’ and ‘other’.

#### Outcomes

The following outcomes were examined: (1) in all adults: cigarette smoking prevalence; (2) in current smokers: mean cigarettes per day (CPD), percentage who smoke within 30 minutes of waking, and high motivation to stop (‘really want and plan to stop within 3 months’) and regular exposure to smoking by others; (3) in past‐year smokers: percentage with a past‐year quit attempt; and (4) in smokers with quit attempts during the past year: percentage not currently smoking and who used cessation support (behavioural, nicotine replacement therapy (NRT) over the counter (OTC), electronic cigarettes (e‐cigarettes) or prescription medication). In this study, CPD and time to first smoke after waking were used as markers of degree of cigarette addiction 24.

#### Potential confounders

Potential confounders, selected a priori, were sex, age, social grade, ‘government office region’ 25 and survey year. Age range was categorized as: 16–24, 25–34, 35–44, 45–54, 55–64 and 65+ years. Social grade was categorized as ‘AB’ (higher and intermediate managerial, administrative or professional managerial, administrative or professional), ‘C1’ (supervisory or clerical and junior managerial, administrative or professional), ‘C2’ (skilled manual workers), ‘D’ (semi‐ and unskilled manual workers) and ‘E’ (casual or lowest‐grade workers, pensioners and others who depend on welfare). Government office regions divide England into the following nine regions: ‘North East’, ‘North West’, ‘Yorkshire and the Humber’, ‘East Midlands’, ‘West Midlands’, ‘East of England’, ‘London’, ‘South East’ and ‘South West’.

### Analysis

Variables were weighted using rim (marginal) weighting to match an English population profile relevant to the time each monthly survey was conducted on the dimensions of age, social grade, region, housing tenure, ethnicity and working status within sex derived from English census data, Office of National Statistics (ONS) mid‐year estimates and other random probability surveys 23. Analyses focused on associations between housing tenure and the smoking and cessation outcomes and were undertaken using SPSS statistical software. Cross‐tabulations were performed to give percentages and linear or logistic regression models were derived to obtain B coefficients or odds ratios (OR) with 95% confidence intervals (CIs) and *P*‐values, depending on whether outcomes were continuous or binary. Outcomes by housing tenure are reported in tabular format with and without adjustment for potential confounders. Missing data were removed on a per‐analysis basis for each outcome. There were no missing data on housing tenure, sex, age, social grade, exposure to cigarette use by others, use of cessation aids or quit success. Missing data on other variables were generally low: government office region 0.1%, motivation to quit 0.2%, time to first smoke after waking 0.4%, CPD 1.0% and past‐year quit attempts 2.6%. The analysis was pre‐registered and SPSS syntax is available on the OpenScienceFramework (https://osf.io/p5zjv/). In addition to our pre‐planned analyses, we performed two sensitivity analyses in which we replicated the adjusted models (i) using a five‐level housing tenure variable (social housing, owned outright, bought on a mortgage, privately rented, other) with social housing as the reference category and (ii) using log‐binomial regression as an alternative to logistic regression, to explore any differences in results.

## Results

### Socio‐demographic characteristics

Sample characteristics are given in Table 1. A total of 8073 (14.0%) of the sample were social housing residents. Those in social housing were more likely to be female, younger and have lower social grade and were more likely to live in London.

**Table 1:** Sample characteristics.

	Total (N = 57 522)		Social housing residents (n = 8073)		Other housing residents (n = 49 449)	
	N	%	n	%	n	%
Female	29 333	51.0	4738	58.7	24 595	49.7
Age (years)						
16–24	8241	14.3	1296	16.1	6945	14.0
25–34	9629	16.7	1649	20.4	7979	16.1
35–44	9532	16.6	1388	17.2	8144	16.5
45–54	9986	17.4	1306	16.2	8679	17.6
55–64	8069	14.0	968	12.0	7101	14.4
65+	12 065	21.0	1465	18.1	10 600	21.4
Social grade						
AB	15 566	27.1	429	5.3	15 136	30.6
C1	15 822	27.5	1309	16.2	14 513	29.4
C2	12 548	21.8	2068	25.6	10 480	21.2
D	8650	15.0	2157	26.7	6493	13.1
E	4936	8.6	2110	26.1	2826	5.7
Government office region						
North East	2877	5.0	574	7.1	2303	4.7
North West	7597	13.2	868	10.8	6729	13.6
Yorkshire and The Humber	5837	10.1	693	8.6	5144	10.4
East Midlands	5031	8.7	796	9.9	4235	8.6
West Midlands	5853	10.2	866	10.7	4987	10.1
East of England	6479	11.3	948	11.8	5531	11.2
London	8560	14.9	1574	19.5	6986	14.1
South East	9333	16.2	964	11.9	8369	16.9
South West	5900	10.3	785	9.7	5115	10.4

### Smoking and cessation outcomes

Social housing residents had almost three times the odds of being a smoker compared with other housing residents (OR = 2.80, 95% CI = 2.66, 2.95, *P* < 0.001; see Table 2) and twice the odds after adjusting for potential confounders (OR = 2.09, 95% CI = 1.98, 2.22, *P* < 0.001).

**Table 2:** Smoking and cessation behaviour in social housing compared to other housing.

			Unadjusted			Adjusted^c^		
	Social housing^a^	Other housing	OR/B^b^	95% Confidence interval	p	OR/B	95% Confidence interval	p
All adults								
% Cigarette smokers	33.8	15.4	2.80	2.66–2.95	< 0.001	2.09	1.98–2.22	< 0.001
Current cigarette smokers								
Cigarettes per day	12.5	10.7	1.82	1.46–2.18	< 0.001	1.09	0.72–1.46	< 0.001
% First smoke within 30 minutes of waking	59.8	42.9	1.98	1.81–2.16	< 0.001	1.63	1.48–1.79	< 0.001
% High motivation to stop	14.2	14.7	0.96	0.84–1.08	0.484	1.04	0.91–1.19	0.553
% Regular exposure to smoking by others	68.9	68.0	1.04	0.95–1.15	0.376	1.08	0.97–1.20	0.142
Past‐year smokers								
% Past year quit attempt	33.0	32.3	1.03	0.94–1.13	0.538	1.14	1.03–1.26	0.011
Past year quit attempt								
% Not currently smoking	11.8	20.4	0.53	0.42–0.66	< 0.001	0.57	0.45–0.72	< 0.001
% Used any cessation support^d^	61.5	56.8	1.22	1.04–1.42	0.012	1.30	1.09–1.54	0.003
% Used behavioural support	3.1	2.5	1.25	0.80–1.95	0.320	1.37	0.83–2.25	0.218
% Used NRT OTC	13.5	12.9	1.06	0.85–1.32	0.590	0.94	0.74–1.20	0.623
% Used e‐cigarettes	35.6	33.3	1.11	0.95–1.29	0.207	1.22	1.02–1.45	0.029
% Used prescription medication	9.3	8.2	1.15	0.89–1.50	0.286	1.24	0.93–1.66	0.142

a Social housing category includes properties rented from local authority and housing association.

b B can be interpreted as the mean (unadjusted/adjusted, as relevant) difference between the social housing and other housing groups.

c OR/B adjusted for sex, age, social grade, government office region and survey year.

d Any cessation support includes behavioural support, nicotine replacement therapy (NRT) bought over‐the‐counter (OTC), e‐cigarettes and prescription medication.

After adjustment, social housing residents smoked more CPD (adjusted mean difference 1.09 cigarettes, 95% CI = 0.72, 1.46, *P* < 0.001) and were more likely to smoke within the first 30 minutes of waking (OR = 1.63, 95% CI = 1.48, 1.79, *P* < 0.001). Both social and other housing residents reported similar regular exposure to smoking by others (OR = 1.08, 95% CI = 0.97, 1.20, *P* = 0.142) and motivation to stop (OR = 1.04, 95% CI = 0.91, 1.19, *P* = 0.553). Social housing residents were more likely to report a quit attempt in the past year (OR = 1.14, 95% CI= 1.03, 1.26, *P* = 0.011) and the use of cessation support (OR = 1.30, 95% CI = 1.09, 1.54, *P* = 0.003). However, they had almost half the odds of stopping smoking successfully (OR = 0.57, 95% CI = 0.45, 0.72, *P* < 0.001).

There was little difference in the pattern of results when housing tenure was analysed as a five‐level variable (Supporting information, Table S1) or when data were analysed using log‐binomial regression (Supporting information, Table S2), although the difference in the rate of use of cessation support became non‐significant in the latter analysis [risk ratio (RR) = 1.11, 95% CI = 0.98, 1.27, *P* **=** 0.110].

## Discussion

Adults living in social housing in England had almost twice the odds of smoking compared with those in other housing tenures, after adjusting for a range of socio‐demographic factors. Among smokers, those in social housing were more addicted to cigarettes. Prevalence of high motivation to stop smoking and regular exposure to smoking by others were similar to that of people in other housing types. Prevalence of quit attempts and use of cessation support within the past year were greater in social housing residents, but success in quitting was much lower.

This study demonstrates that living in social housing is a major independent risk factor for smoking. A location‐based and easily identifiable indicator of smoking hot‐spots could provide the basis for local policy and targeted action to reduce health inequalities. Practically, local authorities in England have control and responsibility for both social housing and public health, including smoking cessation. Authorities could target social housing hot‐spots with localized smoking cessation campaigns and provision of neighbourhood services. Personalized consultation with local communities could broach and build consensus on the sensitive topic of requiring social housing units to be smoke‐free. A complex, multi‐component approach could work synergistically to create cultures of quitting 17, 18. Budgets in local authorities are currently limited by national austerity, but investment in smoking cessation can achieve relatively short‐term returns on investment. A ‘return on investment’ tool provided by the National Institute for Health and Care Excellence can support local commissioners and policymakers to evaluate a portfolio of tobacco control interventions and model economic returns 26. Without action, current disparities are likely to have major health implications for social housing residents.

The most controversial option would be smoke‐free social housing. Findings in the current study are germane to this policy. It is clear that smokers residing in social housing are motivated and attempting to quit smoking, but are more addicted and less likely to succeed in stopping, despite being more likely to use cessation support. A smoke‐free policy to promote quitting among a motivated but unsuccessful group reporting high levels of addiction is more likely to gain traction with politicians and the public. The plan for all public housing to be smoke‐free by autumn 2018 in the United States sets a helpful precedent 19. Action in England may be justified further by explaining how non‐smokers, including children, in multi‐unit housing are more likely to suffer from unknown second‐hand exposure than those living in detached houses 20, 21, which can be reduced by legislation 22. There may be concerns from some quarters concerning the intrusion of rule‐making into the home. A counter‐argument would be that action is required to level the playing field: private landlords routinely insist upon no‐smoking clauses, albeit for self‐interested rather than public health motives (e.g. reduced fire hazard, lower insurance costs and decreased cleaning costs) 15.

The independent association of social housing with smoking status may be partly related to the tendency of smoking to cluster in social networks 16. The finding that social housing residents had both greater cigarette addiction and lower quit success rates is consistent with the literature that smoking dependence is a key determinant of the success rate of cessation 24, 27, 28, 29.

The failure to find evidence of a difference in regular exposure to smoking by other people by housing tenure appears incongruous with there being such a notable difference in smoking prevalence. The result may reflect a ceiling effect on this broad measure of exposure, with the majority of all smokers across housing tenure regularly exposed to smoking by at least one other person. A difference may have emerged had there been an assessment of the number of other smokers to whom respondents were regularly exposed.

The major strengths of this paper are the use of a large representative sample of the population and being the first to provide a detailed characterization of smoking and cessation in social housing in a high‐income country with comprehensive tobacco control.

One limitation of this study is the dichotomization of housing tenure. Social housing and other housing residents have diverse socio‐demographic and socio‐economic backgrounds. However, the models were adjusted at the individual level for differences in several important socio‐demographic confounders. There are additional limitations associated with the use of cross‐sectional survey data, including self‐reported data and non‐biochemically verified status. However, social pressure is less than in the context of the evaluation of an intervention, and there is reason to believe that misreporting is limited in population surveys 30. No data were available on where exposure to smoking by others occurred, so while we found that people living in social housing were more likely to be exposed to smoking by others, whether this exposure occurred in the housing environment is not known. While we adjusted for a range of relevant covariates, there are other factors associated with both housing tenure and smoking behaviour (e.g. depression, stress) that were not assessed in our survey. Another limitation is that the data were collected in England only. While the findings will not necessarily generalize beyond England, they suggest a need to study the issue in other countries. There is international recognition of the need to reduce increasing smoking disparities, and insofar that the finding generalizes, it is possible that smoking cessation policy and interventions relating social housing could be a tractable policy internationally. It would be most sensible to examine countries in a similar position first. England is a high‐income country with extensive tobacco control measures and a smoking prevalence that has declined since the 1970s. A final issue is the assessment of cessation success by asking respondents who had attempted to stop whether they were ‘still not smoking’. This limitation would be serious if the rate of forgetting of failed attempts was associated with housing tenure, and could be assessed in future studies.

## Conclusion

In England, living in social housing is a major independent risk factor for smoking. After adjusting for other important socio‐demographic factors, people in social compared with other housing have twice the odds of smoking. These easily identifiable smoking hot‐spots consist of motivated smokers who find it difficult to stop. Social housing could a key focus for tractable local policy and interventions to reduce smoking‐related health inequalities.

## Declaration of interests

J.B. has received unrestricted research grants from Pfizer relating to the study of smoking cessation; R.W. undertakes research and consultancy and receives fees for speaking from companies that develop and manufacture smoking cessation medications (Pfizer, J&J, McNeil, GSK, Nabi, Novartis and Sanofi‐Aventis); there are no other financial relationships with any organizations that might have an interest in the submitted work in the previous 3 years and there are no other relationships or activities that could appear to have influenced the submitted work.

## Supporting information


**Table S1** Smoking and cessation behaviour in relation to housing tenure.
**Table S2** Smoking and cessation behaviour in social housing compared to other housing: log‐binomial regression models.
