# Supplementary material for: Finding smoking hot‐spots: a cross‐sectional survey of smoking patterns by housing tenure in England
Source: Addiction. 2019 Jan 20;114(5):889–95. doi: 10.1111/add.14544 (PMC6491989; doi:10.1111/add.14544)
Supplement: Supplementary file 1 — Table S1 Smoking and cessation behaviour in relation to housing tenure. Table S2 Smoking and cessation behaviour in social housing compared to other housing: log‐binomial regression models. [file ADD-114-889-s001.docx]

*Supplementary Table 1.* Smoking and cessation behaviour in relation to housing tenure

|  | Social housing |  | Owned outright | | |  | Bought on a mortgage | | |  | Privately rented | | |  | Other housing | | | |
| --- | --- | --- | --- | --- | --- | --- | --- | --- | --- | --- | --- | --- | --- | --- | --- | --- | --- | --- |
|  |  |  | OR | 95% CI | *p* |  | OR | 95% CI | *p* |  | OR | 95% CI | *p* |  |  |  |  |  |
|  | OR |  |  |  |  |  |  |  |  |  |  |  |  |  | OR | 95% CI | *p* |  |
| All adults |  |  |  |  |  |  |  |  |  |  |  |  |  |  |  |  |  |  |
| *% Cigarette smokers* | 1.00 |  | 0.33 | 0.31-0.35 | <0.001 |  | 0.38 | 0.36-0.41 | <0.001 |  | 0.76 | 0.71-0.81 | <0.001 |  | 0.41 | 0.34-0.48 | <0.001 |  |
|  |  |  |  |  |  |  |  |  |  |  |  |  |  |  |  |  |  |  |
| Current cigarette smokers |  |  |  |  |  |  |  |  |  |  |  |  |  |  |  |  |  |  |
| *% First smoke within 30 min of waking* | 1.00 |  | 0.59 | 0.52-0.67 | <0.001 |  | 0.59 | 0.52-0.66 | <0.001 |  | 0.65 | 0.58-0.72 | <0.001 |  | 0.56 | 0.41-0.78 | 0.001 |  |
| *% High motivation to stop* | 1.00 |  | 0.80 | 0.67-0.97 | 0.024 |  | 1.15 | 0.97-1.36 | 0.106 |  | 0.95 | 0.81-1.11 | 0.490 |  | 0.98 | 0.63-1.53 | 0.938 |  |
| *% Regular exposure to smoking by others* | 1.00 |  | 0.81 | 0.71-0.93 | 0.002 |  | 0.86 | 0.75-0.98 | 0.020 |  | 1.06 | 0.94-1.20 | 0.321 |  | 0.79 | 0.56-1.10 | 0.158 |  |
|  |  |  |  |  |  |  |  |  |  |  |  |  |  |  |  |  |  |  |
| Last-year smokers |  |  |  |  |  |  |  |  |  |  |  |  |  |  |  |  |  |  |
| *% Past year quit attempt* | 1.00 |  | 0.78 | 0.68-0.89 | <0.001 |  | 0.96 | 0.84-1.08 | 0.471 |  | 0.88 | 0.79-0.99 | 0.028 |  | 1.21 | 0.89-1.64 | 0.219 |  |
|  |  |  |  |  |  |  |  |  |  |  |  |  |  |  |  |  |  |  |
| Past year quit attempt |  |  |  |  |  |  |  |  |  |  |  |  |  |  |  |  |  |  |
| *% Not currently smoking* | 1.00 |  | 1.89 | 1.39-2.57 | <0.001 |  | 1.95 | 1.47-2.59 | <0.001 |  | 1.63 | 1.25-2.12 | <0.001 |  | 1.63 | 0.89-3.01 | 0.116 |  |
| *% Used any cessation support**** | 1.00 |  | 0.58 | 0.46-0.74 | <0.001 |  | 0.92 | 0.74-1.14 | 0.455 |  | 0.78 | 0.65-0.95 | 0.012 |  | 0.96 | 0.59-1.56 | 0.867 |  |
| *% Used behavioural support* | 1.00 |  | 0.74 | 0.39-1.40 | 0.353 |  | 0.66 | 0.35-1.26 | 0.211 |  | 0.82 | 0.46-1.47 | 0.503 |  | - | - | - |  |
| *% Used NRT OTC* | 1.00 |  | 0.97 | 0.70-1.36 | 0.877 |  | 1.14 | 0.84-1.55 | 0.413 |  | 1.07 | 0.81-1.42 | 0.631 |  | 1.08 | 0.52-2.26 | 0.831 |  |
| *% Used e-cigarettes* | 1.00 |  | 0.71 | 0.55-0.90 | 0.005 |  | 0.99 | 0.80-1.23 | 0.931 |  | 0.79 | 0.65-0.96 | 0.019 |  | 0.87 | 0.52-1.44 | 0.582 |  |
| *% Used prescription medication* | 1.00 |  | 0.57 | 0.38-0.85 | 0.006 |  | 0.79 | 0.55-1.14 | 0.216 |  | 0.93 | 0.67-1.30 | 0.682 |  | 1.54 | 0.72-3.30 | 0.265 |  |

All values are adjusted for sex, age, social grade, government office region, and survey year.

*Supplementary Table 2.* Smoking and cessation behaviour in social housing compared to other housing: log-binomial regression models

|  | Adjusted* | | |
| --- | --- | --- | --- |
|  | RR* | 95% Confidence Interval | *p* |
|  |  |  |  |
| All adults |  |  |  |
| *% Cigarette smokers* | 1.69 | 1.61-1.79 | <0.001 |
|  |  |  |  |
| Current cigarette smokers |  |  |  |
| *% First smoke within 30 min of waking* | 1.24 | 1.15-1.34 | <0.001 |
| *% High motivation to stop* | 1.03 | 0.90-1.17 | 0.709 |
| *% Regular exposure to smoking by others* | 1.03 | 0.96-1.10 | 0.464 |
|  |  |  |  |
| Last-year smokers |  |  |  |
| *% Past year quit attempt* | 1.10 | 1.00-1.20 | 0.048 |
|  |  |  |  |
| Past year quit attempt |  |  |  |
| *% Not currently smoking* | 0.63 | 0.50-0.80 | <0.001 |
| *% Used any cessation support**** | 1.11 | 0.98-1.27 | 0.110 |
| *% Used behavioural support* | 1.41 | 0.88-2.27 | 0.152 |
| *% Used NRT OTC* | 0.94 | 0.75-1.19 | 0.628 |
| *% Used e-cigarettes* | 1.12 | 0.96-1.32 | 0.142 |
| *% Used prescription medication* | 1.25 | 0.94-1.65 | 0.119 |

*Social housing category includes properties rented from local authority and housing association. *RR adjusted for sex, age, social grade, government office region, and survey year. **Relative risk of outcome for individuals living in social housing relative to other tenures (reference category). ***Any cessation support includes behavioural support, nicotine replacement therapy (NRT) bought over-the-counter (OTC), e-cigarettes and prescription medication.
